# The role of maternal age on the risk of preterm birth among singletons and multiples: a retrospective cohort study in Lombardy, Norther Italy

**DOI:** 10.1186/s12884-022-04552-y

**Published:** 2022-03-22

**Authors:** Giovanna Esposito, Paola Agnese Mauri, Sonia Cipriani, Matteo Franchi, Giovanni Corrao, Fabio Parazzini

**Affiliations:** 1 Department of Clinical Sciences and Community Health, University of Milan, 20122 Milan, Italy; 2 Department of Woman, Newborn and Child, University of Milan, Fondazione IRCCS Ca’ Granda Ospedale Maggiore Policlinico, 20122 Milan, Italy; 3 Laboratory of Healthcare Research & Pharmacoepidemiology, Department of Statistics and Quantitative Methods, University of Milano-Bicocca, Milan, Italy; 4 National Centre for Healthcare Research and Pharmacoepidemiology, Milan, Italy

**Keywords:** Preterm birth, Singletons, Multiples, Maternal age

## Abstract

**Background:**

All over the world, especially in the developed countries, maternal age at birth is rising. This study aimed to assess the role of maternal age on the occurrence of preterm birth (PTB) in a large birth cohort of Lombardy Region, Northern Italy.

**Methods:**

This population-based study used data from regional healthcare utilization databases of Lombardy to identify women who delivered between 2007 and 2017. PTBs were defined as births before 37 completed weeks of gestation and considered according to the gestational age (two categories: < 32 weeks and 32 to 36 weeks). Six maternal age groups were defined (< 20, 20–24, 25–29, 30–34, 35–39, ≥40 years). Logistic regression models were fitted to estimate the crude and adjusted odds ratio (aOR) and the corresponding 95% confidence interval (CI) for PTB among different maternal age groups. Analyses were separately performed according to type of pregnancy (singletons and multiples). Reference group was the age group with the lowest frequency of PTB.

**Results:**

Overall, 49,759 (6.6%) PTBs were observed, of which 41,807 were singletons and 7952 were multiples. Rates of PTB were lowest in the women aged 25–29 years among singletons and in the 30–34 years old group among multiples. Our results described a U-shaped association between maternal age and risk of PTB. In particular, the risk of a singleton PTB between 32 and 36 weeks was significantly higher for women aged less than 20 years (aOR = 1.16, CI 95%: 1.04–1.30) and more than 40 years (aOR = 1.62 CI 95%: 1.54–1.70). The highest risk of a multiple delivery between 32 and 36 weeks was observed among women aged less than 25 years and more than 40 years (aOR = 1.79, CI 95%: 1.01–3.17, aOR = 1.47, CI 95%: 1.16–1.85 and aOR = 1.36, CI 95%: 1.19–1.55 respectively for < 20, 20–24 and > 40 age categories). PTB before 32 completed weeks occurred more frequently in the same age categories, except that among multiples no association with advanced maternal age emerged.

**Conclusion:**

Our study suggested that, after adjustment for potential confounders, both advance and young maternal age were associated with an increased risk of PTB.

**Supplementary Information:**

The online version contains supplementary material available at 10.1186/s12884-022-04552-y.

## Introduction

Preterm birth (PTB), defined by the World Health Organization (WHO) as a birth before 37 completed weeks (259 days) of gestation whether singleton or multiple [1], represents the most important factor related to increased risk of fetal and neonatal morbidity and mortality, even in high-income countries. Moreover, a delivery before the 37th week may lead to long-term adverse consequences for offspring health [2].

The aetiology of prematurity is complex: demographic (e.g., low socioeconomic and educational status, too low or too high maternal age), medical, obstetrical, fetal, and environmental aspects concur. Therefore, in most cases the specific mechanism leading to a PTB cannot be established [3].

According to a recent review, the estimated global PTB rate was approximately 10.6% in the 2014, ranged from 13.4% in North Africa to 8.7% in Europe [4].

All over the world, especially in the developed countries, maternal age at birth is rising. Since advanced maternal age has been associated with an increased risk of PTB, an increment in the frequency of PTB could be expected too. Along this line, in Canada, increasing rate was attributable to the advancing maternal age at birth [5].

In any case, the role of maternal age as an independent factor leading to a birth before 37 completed weeks has not yet been fully explained. In literature, some studies investigate influence of maternal age adjusting for obstetrical complications and obtained controversial results [6–9].

Further, in the United States and others industrialized countries, the positive trend in PTB was explained by the rising number of indicated births before the 37th week [10]. Even if more recent evidence from different countries, such as Netherlands and the United States, documented a decline in the singleton PTB [11, 12], among multiples the PTB increased because of the high number of indicated preterm delivery [11]. Moreover, the high number of PTB among multiple gestations achieved by assisted reproductive technologies (ART) represents an important contributor to the overall increase in PTB [2], and singleton pregnancies conceived non spontaneously are also at increased risk of PTB [4, 6].

In Italy, the mean age of women at first birth is the highest in Europe and the proportion of women delaying childbirth beyond 35 years is increasing. Likewise the frequency of ART births was increased of about 12% during the last decade (https://www.istat.it/).

In order to describe the trend of PTB among singletons and multiples and to investigate the independent role of maternal age, we conduced a retrospective population-based cohort study during 2007–2017 in Lombardy, Northern Italy.

## Methods

This is a retrospective cohort study using data retrieved from the regional healthcare utilization (HCU) databases of Lombardy Region between 1st January 2007 and 31st December 2017. The automated system of HCU databases collects several information. For our study, we took into account (i) the archive of residents who receive National Health Service (NHS) assistance including demographic and administrative data, (ii) the database reporting a variety of information about diagnoses and procedures performed on inpatients in public or private hospitals, (iii) the database on outpatient drug prescriptions reimbursable by the NHS, and (iv) the Certificate of Delivery Assistance (CedAP) registry providing detailed information about mother, pregnancy, delivery, and newborn. A deterministic record linkage between the different sources through the unique identification code included in each database allowed to obtain a wide variety of information for each unit of our cohort. As all data are anonymous, ethical approval is not required in Italy.

We identified all the deliveries at 22 to 42 weeks’ gestational age from women beneficiaries of NHS, resident in Lombardy, and aged 13 to 55 years at births. We did not include records of deliveries which did not match to a hospital discharge form including an International Classification of Diseases, 9th revision, Clinical Modification (ICD-9-CM) code or a Diagnosis Related Group (DRG) code related to childbirth and those for which the card of infant could not be linked to the mother’s one. In addition, births were excluded if missing information on mode of conception or on modality of delivery.

The total number of deliveries, in separate strata of singletons and multiples, were obtained. At all stages of the analysis, the groups of singleton and multiple births were considered separately.

The PTB rate was calculated dividing the observed number of PTBs by the total number of deliveries, all over the time of the study and for the single years.

PTBs and at term births were compared for maternal age, socio-demographic characteristics (i.e., nationality, marital status, education, and employment), type of conception (i.e., spontaneous and ART), gestational diabetes, and hypertension. Differences on categorical variables were tested by using the chi-squared test. Differences on maternal age between the two groups were tested by using the t-test for independent samples.

Six categories of maternal age were defined (< 20, 20–24, 25–29, 30–34, 35–39, ≥40 years) and were compared on the basis of the baseline covariates mentioned above.

Common preterm classification system is based on gestational age sub-groups, such as extremely preterm (< 28 weeks), very preterm (28 to 31 completed weeks), and moderate to late preterm (32 to 36 weeks) [13]. In our analysis, we decided to join the first two categories in a single one, because the birth before the 28th week represents less than 5% of all births before 37 completed weeks. PTB could also be categorized by its clinical presentation: medically induced and spontaneous preterm labor.

Logistic regression models were fitted to estimate the odds ratio (OR) and the corresponding 95% confidence interval (CI) for PTB in strata of gestational age (two categories: births between 32 and 36 weeks and births before 32 completed weeks) and mode of labor (two categories: induced and spontaneous) among different maternal age groups. Adjusted OR (aOR) for nationality, marital status, education, employment, type of conception, gestational diabetes and hypertension disorders was also estimated. Reference group was the age group with the lowest frequency of PTB. Results were considered statistically significant when two-tailed *p*-value was less than 0.05.

Finally, in order to consider the advancing maternal age in the last years, we estimated the proportion of PTB that could be attributable to advanced maternal age (≥35 years) in the entire population according to calendar years. We calculated the population attributable fraction (PAF) using this formula: [(risk of PTB in all women - risk of PTB in women aged less 35 years)/risk of PTB in all women]*100.

All analyses were performed using the Statistical Analysis System Software (version 9.4; SAS Institute, Cary, NC, USA).

## Results

A total of 915,193 deliveries were registered in the CedAP database of the Lombardy from the 1st January 2007 to the 31st December 2017. We excluded 7685 records because did not match to a hospital discharge form related to childbirth, 143,788 records of women not resident in Lombardy, 839 records because the mother was younger than 13 years or older than 55 years of age at delivery, 2891 records because the gestational age was less than 22 weeks or more than 42 weeks, 458 records because the infant could not be linked to the mother, and 5147 records because information on mode of conception or on mode of delivery was missing.

Thus, we obtained a final cohort including 754,385 deliveries. Among these, during the study period, 49,759 (6.6%) PTBs were observed.

Out of the total deliveries, 741,150 (98.2%) were singletons and 13,235 (1.8%) were multiples. During the period of the study, the proportion of multiple births was not constant over the years; at first multiples increased, but in the last years appeared decreasing.

Among singleton births, 41,807 (5.6%) PTBs were identified and distributed by strata of maternal age as followed: 434 aged less than 20 years, 2289 aged 20–24 years, 7235 aged 25–29 years, 14,103 aged 30–34 years, 14,719 aged 35–39 years and 3027 aged more than 40 years. Whereas, among multiples, 7952 (60.1%) births occurred before the 37th week and 42 women were aged less than 20 years, 254 aged 20–24 years, 1105 aged 25–29 years, 2668 aged 30–34 years, 2972 aged 35–39 years and 911 aged more than 40 years.

No significant trend in the total number of singleton and multiple PTBs, regardless induced or spontaneous, emerged over calendar year at birth (Fig. S1 and Fig. S2).

The distribution of maternal characteristics of at term births and PTBs among singleton and multiple deliveries are shown in Supplementary material (Table S1 and Table S2). Singleton PTBs were more frequent among older women (*p* < 0.0001). Others maternal demographic characteristics (i.e., low educational and employment status, single marital status, and foreign nationality) and complication during pregnancy (i.e., hypertension disorders and gestational diabetes) were more common among singleton PTBs (*p*-value< 0.0001). Instead, multiple PTBs were more frequent among older women, but also among too young women (*p* < 0.0001). Moreover, no significant differences emerged regarding other maternal characteristics. Regarding the mode of conception, a higher number of births after ART was observed among multiple births compared with singletons (respectively about 28 and 2%). Among multiple births, no significant differences emerged on the medically conceiving comparing at term and PTBs (*p*-value = 0.0613). Instead, among singletons, PTB occurred more frequently when pregnancy was obtained with ART.

The covariates at baseline by maternal age group are presented in Table 1 and Table 2. Younger women of both two subgroups (multiple and singleton births) were more commonly not Italian, lower educated and employed, and not married if compared with older (*p* < 0.0001). Pregnancies of advanced maternal age mothers were more frequently complicated by hypertension disorders and gestational diabetes; this finding was more evident among singleton births (*p* < 0.0001). In general, the rate of ART was lowest in women aged less than 30 years (*p* < 0.0001).

**Table 1:** Baseline characteristics according to maternal age group among singleton preterm births. Lombardy, Italy, 2007–2017

	Maternal age group						
	< 20 (*N* = 434)	20–24 (*N* = 2289)	25–29 (*N* = 7235)	30–34 (*N* = 14,103)	35–39 (*N* = 14,719)	≥40 (*N* = 3027)	*p*-value
*Year of birth*							
2007	37 (8.5)	152 (6.6)	641 (8.9)	1337 (9.5)	1174 (8.0)	175 (5.8)	< 0.0001
2008	32 (7.4)	222 (9.70)	720 (10.0)	1444 (10.2)	1396 (9.5)	202 (6.7)	
2009	40 (9.2)	194 (8.5)	690 (9.5)	1443 (10.2)	1350 (9.2)	234 (7.7)	
2010	37 (8.5)	194 (8.5)	640 (8.9)	1240 (8.8)	1.331 (9.0)	241 (8.0)	
2011	48 (11.1)	183 (8.0)	670 (9.3)	1306 (9.3)	1.329 (9.0)	254 (8.4)	
2012	51 (11.8)	235 (10.3)	702 (9.7)	1.342 (9.5)	1474 (10.0)	284 (9.4)	
2013	37 (8.5)	239 (10.4)	642 (8.9)	1241 (8.8)	1358 (9.2)	306 (10.1)	
2014	46 (10.6)	217 (9.5)	646 (8.9)	1229 (8.7)	1351 (9.2)	333 (11.0)	
2015	37 (8.5)	201 (8.8)	620 (8.6)	1225 (8.7)	1313 (8.9)	310 (10.2)	
2016	34 (7.8)	220 (9.6)	669 (9.3)	1205 (8.5)	1355 (9.2)	342 (11.3)	
2017	35 (8.1)	232 (10.1)	595 (8.2)	1091 (7.7)	1288 (8.8)	346 (11.4)	
*Maternal citizenship*							
Italian	293 (67.5)	1490 (65.1)	5379 (74.4)	11,454 (81.2)	12,235 (83.1)	2547 (84.1)	< 0.0001
Not Italian	141 (32.5)	799 (34.9)	1856 (25.6)	2649 (18.8)	2484 (16.9)	480 (15.9)	
*Marital status* ^a^							
Married	55 (13.1)	911 (41.2)	4524 (64.2)	9778 (71.2)	9978 (69.9)	1915 (65.1)	< 0.0001
Not married	364 (86.9)	1300 (58.8)	2518 (35.8)	3947 (28.8)	4305 (30.1)	1027 (34.9)	
*Maternal education* ^b^							
Middle school	332 (77.2)	1144 (50.4)	2475 (34.3)	3561 (25.4)	3531 (24.1)	779 (25.8)	< 0.0001
High school	94 (21.9)	1043 (46.0)	3559 (49.4)	6526 (46.5)	6465 (44.2)	1303 (43.2)	
University	4 (0.9)	83 (3.6)	1176 (16.3)	3951 (28.2)	4647 (31.7)	938 (31.0)	
*Maternal employment*							
Employed	48 (11.1)	918 (40.1)	4846 (67.0)	10,354 (77.7)	11,522 (78.3)	2305 (76.2)	< 0.0001
Not employed	386 (88.9)	1371 (59.9)	2388 (33.0)	3148 (22.3)	3196 (21.7)	722 (23.9)	
*Mode of conception*							
Spontaneous	433 (99.8)	2284 (99.8)	7150 (98.8)	13,703 (97.2)	13,932 (94.7)	2601 (85.9)	< 0.0001
Non spontaneous	1 (0.2)	5 (0.2)	85 (1.2)	400 (2.8)	787 (5.3)	426 (14.1)	
*Diabetes*							
No	433 (99.8)	2277 (99.5)	7162 (99.0)	13,948 (98.9)	14,493 (98.5)	2943 (97.2)	< 0.0001
Yes	1 (0.2)	12 (0.5)	73 (1.0)	155 (1.1)	226 (1.5)	84 (2.7)	
*Hypertension*							
No	430 (99.1)	2263 (98.9)	7068 (97.7)	13,763 (97.6)	14,300 (97.1)	2893 (95.6)	< 0.0001
Yes	4 (0.9)	26 (1.1)	167 (2.3)	340 (2.4)	419 (2.9)	134 (4.4)	

^a^Not included 1185 missing data

^b^Not included 196 missing data

**Table 2:** Baseline characteristics according to class of maternal age (years) among multiple preterm births. Lombardy, Italy, 2007–2017

	Maternal age group						
	< 20 (*N* = 42)	20–24 (*N* = 254)	25–29 (*N* = 1105)	30–34 (*N* = 2668)	35–39 (*N* = 2972)	≥40 (*N* = 911)	*p*-value
*Year of birth*							
2007	3 (7.1)	19 (7.5)	96 (8.7)	229 (8.6)	197 (6.6)	26 (2.9)	< 0.0001
2008	3 (7.1)	22 (8.7)	106 (9.6)	272 (10.2)	235 (7.9)	32 (3.5)	
2009	4 (9.5)	19 (7.5)	89 (8.1)	276 (10.3)	279 (9.4)	66 (7.24)	
2010	3 (7.1)	17 (6.7)	92 (8.3)	242 (9.1)	271 (9.1)	72 (7.9)	
2011	6 (14.3)	22 (8.7)	126 (11.4)	249 (9.3)	297 (10.0)	77 (8.45)	
2012	1 (2.4)	32 (12.6)	108 (9.8)	230 (8.6)	270 (9.1)	97 (8.5)	
2013	6 (14.3)	20 (7.9)	121 (11.0)	270 (10.1)	298 (10.0)	95 (10.4)	
2014	5 (11.9)	31 (12.2)	99 (9.0)	257 (9.6)	297 (10.0)	131 (14.4)	
2015	7 (16.7)	23 (9.1)	80 (7.2)	202 (7.6)	288 (9.7)	114 (12.5)	
2016	2 (4.8)	28 (11.0)	99 (9.0)	220 (8.3)	278 (9.4)	109 (12.0)	
2017	2 (4.8)	21 (8.3)	89 (8.1)	221 (8.3)	262 (8.8)	92 (10.1)	
*Maternal citizenship*							
Italian	28 (66.7)	171 (67.3)	862 (78.0)	2265 (84.9)	2610 (87.8)	820 (90.0)	< 0.0001
Not Italian	14 (33.3)	83 (32.7)	243 (22.0)	403 (15.1)	362 (12.2)	91 (10.0)	
*Marital status* ^a^							
Married	7 (17.1)	110 (44.00)	712 (66.1)	1991 (76.8)	2134 (73.3)	560 (63.0)	< 0.0001
Not married	34 (82.9)	140 (56.00)	366 (33.9)	600 (23.2)	776 (26.7)	329 (37.0)	
*Maternal education* ^b^							
Middle school	32 (78.1)	123 (48.4)	368 (33.4)	528 (19.9)	545 (18.4)	145 (15.9)	< 0.0001
High school	8 (19.5)	116 (45.7)	531 (48.2)	1218 (45.8)	1237 (41.7)	387 (42.5)	
University	1 (2.5)	15 (5.9)	202 (18.4)	912 (34.3)	1183 (39.9)	378 (41.5)	
*Maternal employment*							
Employed	4 (9.5)	102 (40.2)	790 (71.5)	2143 (80.3)	2470 (83.1)	782 (85.8)	< 0.0001
Not employed	38 (90.5)	152 (59.8)	315 (28.5)	525 (19.7)	502 (16.9)	129 (14.2)	
*Mode of conception*							
Spontaneous	42 (100.0)	246 (96.9)	963 (87.2)	2061 (77.3)	1958 (65.9)	333 (36.6)	< 0.0001
Non spontaneous	0 (0.0)	8 (3.1)	142 (12.8)	607 (2.7)	1014 (34.1)	578 (63.5)	
*Diabetes*							
No	42 (100.0)	253 (99.6)	1099 (99.5)	2637 (98.8)	2938 (98.9)	899 (98.7)	0.3693
Yes	0 (0.0)	1 (0.4)	6 (0.5)	31 (1.2)	34 (1.1)	12 (1.3)	
*Hypertension*							
No	38 (90.5)	250 (98.4)	1072 (97.0)	2601 (97.5)	2893 (97.3)	870 (95.5)	0.0022
Yes	4 (9.5)	4 (1.6)	33 (3.0)	67 (2.5)	79 (2.7)	41 (4.5)	

^a^Not included 193 missing data

^b^ Not included 23 missing data

Over the entire period of study, average maternal age of all births (at term and preterm, multiples and singletons) increased (data not shown).

**Fig. 1:**
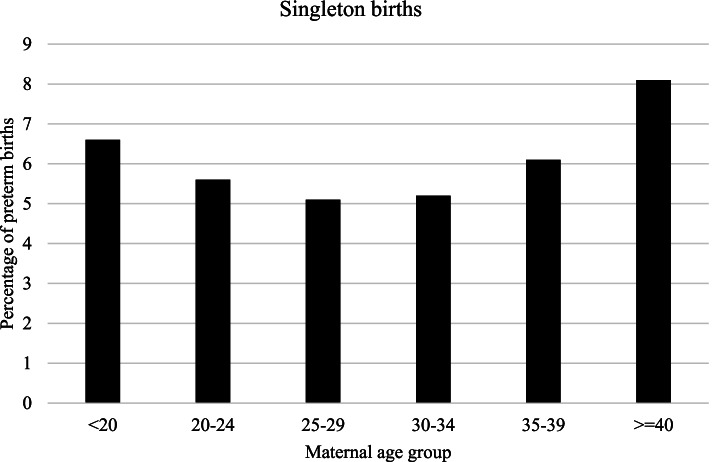
Preterm birth according to maternal age group among singletons. Lombardy, Italy, 2007–2017

**Fig. 2:**
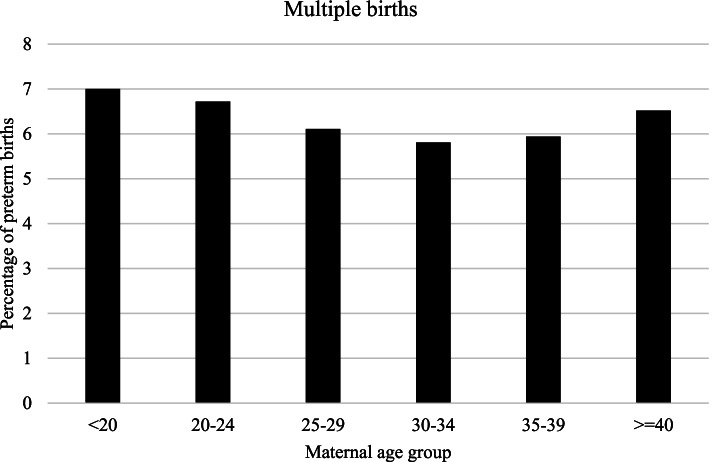
Preterm birth according to maternal age group among multiples. Lombardy, Italy, 2007–2017

In the Fig. 1 and Fig. 2 rates of singleton and multiple PTBs are represented by age categories. Rates of singleton PTB were lowest in the women aged 25–29 years and highest in the over 40 years old group (5.0 and 8.1% respectively). Among multiples, the lowest frequency of PTB was observed in the 30–34 years group and the highest in the group of youngest (58.1 and 70.0% respectively).

**Fig. 3:**
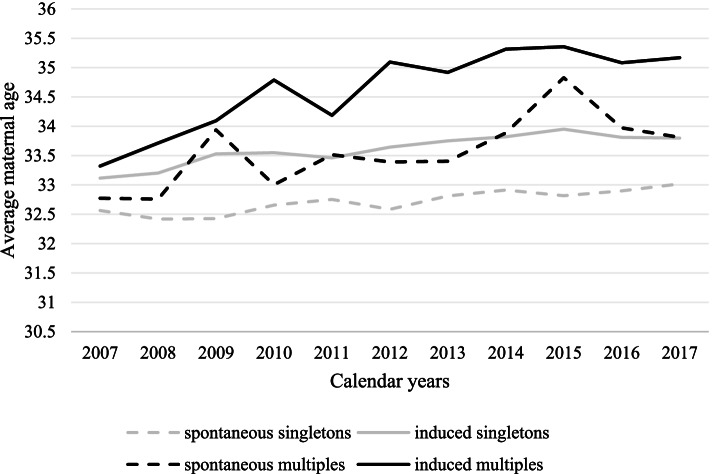
Average maternal age according to mode of labor among singleton and multiple preterm births. Lombardy, Italy, 2007–2017

In particular, a stronger increasing of average maternal age was observed among multiple PTBs when compared with singleton ones. Overall, mean mothers’ age was higher among induced PTB, regardless type of pregnancy (Fig. 3).

Crude and adjusted OR for PTB strata by gestational age are reported in Table 3 and Table 4. Among singleton births, the risk of a PTB between 32 and 36 weeks was significantly higher for women aged less than 20 years (aOR = 1.16, CI 95%: 1.04–1.30) and more than 35 years when compared with women aged 25–29 years. The risk increased consistently for women over 40 years (aOR = 1.62 CI 95%: 1.54–1.70). Singleton PTB before 32 completed weeks occurred more frequently in the same age categories; aORs were respectively 1.50 (CI 95%: 1.19–1.92) and 1.66 (CI 95%: 1.47–1.87) for the youngest and the oldest women. Among multiple births, the risk of a delivery between 32and 36 weeks was significantly higher for young women aged less than 25 years compared with reference group (30–34 years) (aOR = 1.79, CI 95%: 1.01–3.17 and aOR = 1.47, CI 95%: 1.16–1.85 respectively for < 20 and 20–24 age categories). Increased risk was observed also for women over 40 years, although more slightly (aOR = 1.36, CI 95%: 1.19–1.55). Extreme multiple PTBs were more common in women aged 20 to 29 years (aOR = 1.80, CI 95%: 1.23–2.63 and aOR = 1.39, CI 95%: 1.13–1.70 respectively for 20–24 and 25–29 age categories) comparing with reference group. No association between a multiple birth before 32 completed weeks and advanced maternal age was observed.

**Table 3:** Risk of preterm birth strata by gestational age (weeks) according to maternal age (years) among singletons. Lombardy, Italy, 2007–2017

	Maternal age group						
	Overall	< 20	20–24	25–29	30–34	35–39	≥40
	741,150	6580	40,806	143,333	272,934	240,326	37,171
*32–36 w*							
N (%)	36,365 (4.9)	359 (5.5)	1939 (4.8)	6287 (4.4)	12,336 (4.5)	12,821 (5.3)	2623 (7.1)
OR		1.26 (1.13–1.41)	1.09 (1.03–1.15)	1.00	1.03 (1.00–1.06)	1.23 (1.19–1.27)	1.66 (1.59–1.74)
aOR		1.16 (1.04–1.30)	1.03 (0.98–1.09)	1.00	1.08 (1.04–1.10)	1.27 (1.23–1.31)	1.62 (1.54–1.70)
*< 32 w*							
N (%)	5442 (0.7)	75 (1.1)	350 (0.9)	948 (0.7)	1767 (0.6)	1898 (0.8)	404 (1.1)
OR		1.75 (1.38–2.22)	1.31 (1.15–1.48)	1.00	0.98 (0.91–1.06)	1.21 (1.12–1.31)	1.70 (1.51–1.91)
aOR		1.50 (1.19–1.92)	1.18 (1.04–1.34)	1.00	1.06 (0.98–1.15)	1.30 (1.20–1.40)	1.66 (1.47–1.87)

**Table 4:** Risk of preterm birth strata by gestational age (weeks) according to maternal age (years) among multiples. Lombardy, Italy, 2007–2017

	Maternal age group						
	Overall	< 20	20–24	25–29	30–34	35–39	≥40
	13,235	60	378	1809	4589	5002	1397
*32–36 w*							
N (%)	6884 (51.7)	38 (63.3)	213 (56.3)	924 (51.1)	2312 (50.4)	2597 (51.9)	800 (57.3)
OR		1.75 (1.00–3.08)	1.43 (1.14–1.80)	1.09 (0.97–1.22)	1.00	1.06 (0.98–1.16)	1.37 (1.20–1.55)
aOR		1.79 (1.01–3.17)	1.47 (1.16–1.85)	1.11 (0.98–1.24)	1.00	1.06 (0.98–1.16)	1.36 (1.19–1.55)
*< 32 w*							
N (%)	1068 (8.0)	4 (6.7)	41 (10.8)	181 (10.0)	356 (7.8)	375 (7.5)	111 (7.9)
OR		1.20 (0.40–3.57)	1.78 (1.23–2.56)	1.39 (1.14–1.69)	1.00	1.00 (0.85–1.17)	1.23 (0.97–1.56)
aOR		0.83 (0.24–2.86)	1.80 (1.23–2.63)	1.39 (1.13–1.70)	1.00	0.97 (0.83–1.14)	1.11 (0.87–1.41)

PAF of PTB for advanced maternal age according to calendar years is reported in Supplementary material (Table S3). The proportion of PTB attributable to delayed childbearing beyond 35 year increased from 6.6 to 12.2% from 2007 to 2017.

With regard the modality of labor, Table 5 provide ORs for spontaneous and induced PTB among singletons and multiples according to mother’s age. Among singleton births, the risk of a spontaneous PTB was higher for women aged less than 20 years (aOR = 1.38, CI 95%: 1.22–1.57) and more than 40 years (aOR = 1.22, CI 95%: 1.15–1.29) when compared with women aged 25–29 years. Women aged 20 to 34 years appeared experimenting the same risk of a spontaneous labor before 37 completed weeks. No association between young maternal age and induced PTB was observed. The risk of induction of labor before the term of pregnancy increased with increasing maternal age, being about 2-fold higher for mothers age more than 40 years (aOR = 1.96, CI 95%: 1.86–2.08) when compared with mothers aged 25–29 years. Among multiple births, an inverse association between advanced maternal age (≥35 years) and spontaneous PTB emerged (aOR = 0.88, CI 95%: 0.79–0.97 and aOR = 0.72, CI 95%: 0.63–0.83 respectively for 35–39 and ≥ 40 age categories). Instead, among very young mothers (< 20 years) spontaneous labor before the term of pregnancy was more frequent (aOR = 1.76, CI 95%: 1.02–3.10). Induced PTBs were more common among both younger and older women.

**Table 5:** Risk of preterm birth according to maternal age (years) and mode of labor among singletons and multiples. Lombardy, Italy, 2007–2017

	Maternal age group					
	< 20	20–24	25–29	30–34	35–39	≥40
Singletons ^a^						
*Spontaneous*						
N (%)	283 (4.3)	1285 (3.2)	3973 (2.8)	7410 (2.7)	6344 (2.9)	1978 (3.3)
OR	1.57 (1.39–1.78)	1.14 (1.07–1.22)	1.00	0.98 (0.94–1.02)	1.05 (1.01–1.10)	1.20 (1.14–1.27)
aOR	1.38 (1.22–1.57)	1.05 (0.99–1.13)	1.00	1.03 (0.99–1.07)	1.10 (1.06–1.15)	1.22 (1.15–1.29)
*Induced*						
N (%)	146 (2.2)	959 (2.4)	3105 (2.2)	6430 (2.4)	6444 (3.0)	2552 (4.3)
OR	1.02 (0.86- 1.21)	1.09 (1.01–1.17)	1.00	1.09 (1.04–1.14)	1.38 (1.32–1.44)	2.02 (1.92–2.13)
aOR	0.90 (0.76–1.07)	1.00 (0.93–1.08)	1.00	1.15 (1.10–1.20)	1.43 (1.37–1.50)	1.96 (1.86–2.08)
Multiples ^b^						
*Spontaneous*						
N (%)	23 (38.3)	96 (26.5)	456 (25.7)	1041 (23.3)	909 (20.9)	338 (17.8)
OR	2.05 (1.21–3.47)	1.19 (0.93–1.51)	1.14 (1.00–1.29)	1.00	0.87 (0.79–0.96)	0.72 (0.63–0.82)
aOR	1.76 (1.02–3.10)	1.13 (0.87–1.45)	1.12 (0.98–1.28)	1.00	0.88 (0.79–0.97)	0.72 (0.63–0.83)
*Induced*						
N (%)	19 (31.7)	146 (40.2)	624 (35.1)	1544 (34.5)	1657 (38.0)	856 (45.2)
OR	0.88 (0.51–1.52)	1.28 (1.03–1.60)	1.03 (0.92–1.15)	1.00	1.17 (1.07–1.27)	1.57 (1.40–1.75)
aOR	1.01 (0.58–1.78)	1.35 (1.08–1.71)	1.05 (0.93–1.18)	1.00	1.15 (1.05–1.26)	1.48 (1.32–1.66)

^a^Not included 6557 missing data

^b^Not included 306 missing data

## Discussion

The current population-based study suggested that both advanced and young maternal age were associated with an increased risk of PTB, even after adjustment for potential confounders. Therefore, mothers’ age may represent an independent factor leading to a birth before 37 completed weeks. The lowest frequency of PTB was found in mothers aged 25–29 years for singleton births and in mothers aged 30–34 years for multiple ones. Average maternal age was higher among induced deliveries rather than spontaneous, regardless the type of pregnancy.

Our results are consistent with previous evidence. Two cohort studies, conducted in Northern Europe and Canada, included singleton births and described a U-shaped association between maternal age and risk of PTB [8, 14]. In particular, in our analysis this finding was valid for all births, independently by the type of pregnancy, but was more evident among singletons rather than multiples.

In our cohort, younger women of both two subgroups (multiple and singleton births) were more frequently lower educated and employed, foreign, and not married if compared with older ones. A recent cohort study conducted in Lombardy revealed that high-level educated mothers had about 20% decreased risk of PTB and the unemployed, unmarried, and foreign status represented predisposing factors for some adverse perinatal outcomes, including a birth before the term of pregnancy [15]. To support, a meta-analysis involving several European countries reported a 48% risk excess of PTB related to low maternal education [16]. Regarding marital status, according to other sources, PTB was increased not only among unmarried, but especially among cohabitating and single mothers [17]. Instead, literature is controversial about the independent role of unemployment on the risk of PTB due to the largely association with social disadvantage and unfavorable health behaviors representing risk factors of PTB [18]. Moreover, not all studies related the migrant status to PTB and adverse neonatal outcomes, according to the integration policies of the host countries [19]. In addition, in a large population study conducted in a multicultural country as the United States [20], the rates of PTB differed substantially by ethnicity, reaching its peak in non-Hispanic black mothers.

Different mechanisms are involved in the increasing risk of PTB for younger and older women. In our cohort, pregnancies in advanced age women were more commonly complicated by hypertension disorders and gestational diabetes and the rate of ART was more highly prevalent in older mothers. Hypertension and diabetes are widely related to PTB [21, 22]. Furthermore, recent data reported a 50–60% increased risk of PTB in medically conceptions when compared with spontaneous ones [23]. As previous found [14], no confounding effect of socio-economic condition emerged among older mothers. Since all over the world, especially in the developed countries, delayed childbearing beyond 35 years is wide spread and is constantly increasing, we estimated the proportion of PTB that could be attributable to advanced maternal age, reporting a continuous increment during the entire study period from 6.6 to 12.2%. An American study reported that in 2005–2006 the attributable faction of PTB for advanced maternal age was 1.1% in the United States and 6.2% in Canada [24]. To our knowledge, no previous study has investigated how the risk of PTB has changed over the time in relation to the advancing maternal age.

We conduced our analyses stratifying by multiple and singleton births. The risk to develop a maternal or fetal condition leading to a birth before 37 completed weeks is much higher in multiples than in singletons. In the United States a 12-fold increased risk was observed, the rate of PTB was about 57% in multiples vs 10% in singletons [25]. The discrepancies detected are probably due to different pathophysiologic mechanisms of PTB among multiple pregnancies, such as intrauterine infection or inflammation, cervical insufficiency, uterine overdistension, uterine ischemia [26]. In our cohort, multiple PTB were more frequent among older women, probably also due to the higher occurrence of medically conceived pregnancies among this age category.

Finally, we analyzed the modality of labor. The independent role of maternal age on the mode of labor in PTB has been discussed in the literature, showing some controversial findings. A large cohort study from the United Kingdom reported that advancing maternal age was associated with an increased risk of iatrogenic, but not spontaneous, early preterm delivery, recognizing as main indications pre-eclampsia and intrauterine growth restriction, frequent conditions in advanced maternal age [27]. Another recent study based on a large cohort [8] suggested that both spontaneous and iatrogenic preterm labor was more frequent among older women. According to other authors, the risk of iatrogenic preterm delivery increased with maternal age, independently of other confounders (e.g., BMI, education level, parity, method of conception, and pre-existing disease), especially for women aged more than 35 [28, 29]. In our study, among multiple births, advanced age appeared to be a protective factor for spontaneous PTB, conceivably because the high occurrence of indications leading to an elective delivery before the onset of spontaneous labor. Concerning younger mothers, the PTB was mainly spontaneous, no associations between young age and risk of iatrogenic PTB emerged.

In a broader view, the widespread increase in the use of obstetric interventive practices, such as iatrogenic birth at any gestational age, causes concern as interventions developed in response to specific medical conditions may involve adverse effects when used routinely or improperly. The advanced maternal age, particularly in nulliparous women, has been suggested to be a cause of the increment of caesarian sections [30]. This observation may reflect the proneness to a more medicalized approach by the clinicians to an advanced maternal age pregnancy, adopting a lowered treatment threshold for interventions [31, 32] as the pregnancy tends to be “precious” and at “high risk for medical-legal issues” [33].

The concept of medicalization represents a process where a physiological condition is considered as a pathological condition and treated as such. In this contest, the fact that iatrogenic childbirth could be preterm is an aggravating element. However, another recent study based on a large cohort [8] suggested that both spontaneous and iatrogenic preterm labor was more frequent among older women. In our cohort, among singleton pregnancies, even if the risk of induced PTB was higher, we observed also an increased risk of spontaneous ones. This suggests that a careful evaluation of each individual case is always necessary and, in general, physicians should actively educate women that there is a real danger of delayed childbearing for both mother and offspring [34, 35].

The major strengths of this study are the size of the cohort with more of 750,000 births and the wide sources of data over a span of 10 years.

This study has some weaknesses. First, data on well-recognized factors related to PTB, such as body mass index or lifestyle habits (e.g., smoking, alcohol intake, physical activity), were not available in administrative sources analyzed. For example, regarding smoking that is a major risk factor for PTB, it has been reported a decreasing trend in smoking among women of reproductive age (https://www.istat.it/).

Second, the modality of labor could be misclassified. In particular, PTBs by elective cesarean section secondary to preterm premature rupture of membranes were classified as an iatrogenic labor due to the modality of delivery chosen, even if a spontaneous labor was potentially beginning.

## Conclusion

In our large birth cohort, maternal age has been suggested to be independently associated with singleton and multiple PTB, even after adjustment for selected potential confounders. Both younger and older women had a higher risk of PTB.

## Supplementary Information

**Additional file 1.**

## Data Availability

The data that support the findings of this study are available from Lombardy Region, but restrictions apply to the availability of these data which were used under license for the current study, and so are not publicly available. Data are however available from the authors upon reasonable request and with permission of Lombardy Region.
