# Correction to: The role of maternal age on the risk of preterm birth among singletons and multiples: a retrospective cohort study in Lombardy, Northern Italy

**DOI:** 10.1186/s12884-022-04695-y

**Published:** 2022-04-28

**Authors:** Giovanna Esposito, Paola Agnese Mauri, Sonia Cipriani, Matteo Franchi, Giovanni Corrao, Fabio Parazzini

**Affiliations:** 1 Department of Clinical Sciences and Community Health, University of Milan, 20122 Milan, Italy; 2 Department of Woman, Newborn and Child, Universityof Milan, Fondazione IRCCS Ca’ Granda Ospedale Maggiore Policlinico, 20122 Milan, Italy; 3 Laboratory of Healthcare Research & Pharmacoepidemiology, Department of Statistics and Quantitative Methods, University of Milano-Bicocca, Milan, Italy; 4 National Centre for Healthcare Research and Pharmacoepidemiology, Milan, Italy

**Correction to: BMC Pregnancy Childbirth 22, 234 (2022)**

**https://doi.org/10.1186/s12884-022-04552-y**

Following publication of the original article [1], the author reported that there is an error in the title: Norther instead of Northern. Correct article title is “The role of maternal age on the risk of preterm birth among singletons and multiples: a retrospective cohort study in Lombardy, Northern Italy”.

The original article [1] has been corrected.
