# Supplementary material for: The role of maternal age on the risk of preterm birth among singletons and multiples: a retrospective cohort study in Lombardy, Norther Italy
Source: BMC Pregnancy Childbirth. 2022 Mar 22;22:234. doi: 10.1186/s12884-022-04552-y (PMC8941739; doi:10.1186/s12884-022-04552-y)
Supplement: Supplementary file 1 — Additional file 1. [file 12884_2022_4552_MOESM1_ESM.docx]

**Table S1.** Baseline characteristics in the cohort of preterm births and at term singleton births. Lombardy, Italy, 2007-2017.

|  | Reference At Term Births (N=699,343) | PTB (N=41,807) | p-value |
| --- | --- | --- | --- |
| *Year of birth* | |  | |
| 2007 | 59,895 (8.6) | 3,516 (8.4) | 0.0002 |
| 2008 | 66,268 (9.5) | 4,016 (9.6) |  |
| 2009 | 65,199 (9.3) | 3,951 (9.5) |  |
| 2010 | 62,973 (9.0) | 3,683 (8.8) |  |
| 2011 | 67,281 (9.6) | 3,790 (9.1) |  |
| 2012 | 66,601 (9.5) | 4,088 (9.8) |  |
| 2013 | 64,578 (9.2) | 3,823 (9.1) |  |
| 2014 | 64,146 (9.2) | 3,822 (9.1) |  |
| 2015 | 62,778 (9.0) | 3,706 (8.9) |  |
| 2016 | 60,321 (8.6) | 3,825 (9.2) |  |
| 2017 | 59,303 (8.5) | 3,587 (8.6) |  |
| *Maternal age (year****s*)** | |  | |
| <20 | 6,146 (5.5) | 434 (1.04) | <0.0001 |
| 20-24 | 38,517 (5.5) | 2,289 (5.5) |  |
| 25-29 | 136,098 (19.5) | 7,235 (17.3) |  |
| 30-34 | 258,831 (37.0) | 14,103 (33.7) |  |
| 35-39 | 225,607 (32.3) | 14,719 (35.2) |  |
| ≥40 | 34,144 (4.9) | 3,027 (7.2) |  |
| *Mean age (year****s)*** | 32.61 5.07 | 33.15 5.37 | <0.0001 |
| *Maternal citizenship* | |  | |
| Italian | 579,050 (82.8) | 33,398 (79.9) | <0.0001 |
| Not Italian | 120,293 (17.2) | 8,409 (20.1) |  |
| *Marital status* ^a^ | |  | |
| Married | 469,813 (68.7) | 27,161 (66.9) | <0.0001 |
| Not married | 213,902 (31.3) | 13,461 (33.1) |  |
| *Maternal education* ^b^ | |  | |
| Middle school | 170,308 (24.5) | 11,822 (28.4) | <0.0001 |
| High school | 318,683 (45.8) | 18,990 (45.6) |  |
| University | 207,106 (29.8) | 10,799 (26.0) |  |
| *Maternal employment* ^c^ | |  | |
| Employed | 527,807 (75.5) | 30,593 (73.2) | <0.0001 |
| Not employed | 171,486 (24.5) | 11,211 (26.8) |  |
| *Mode of conception* | |  | |
| Spontaneous | 684,722 (97.9) | 40,103 (95.9) | <0.0001 |
| Non spontaneous | 14,621 (2.1) | 1,704 (4.1) |  |
| *Diabetes* |  |  |  |
| No | 693,755 (99.2) | 41,256 (98.7) | <0.0001 |
| Yes | 5,588 (0.8) | 551 (1.3) |  |
| *Hypertension* |  |  |  |
| No | 694,940 (99.4) | 40,717 (97.4) | <0.0001 |
| Yes | 4,403 (0.6) | 1,090 (2.6) |  |

^a^ Not included 16,813 missing data ^b^ Not included 3,442 missing data ^c^ Not included 53 missing data

**Table S2.** Baseline characteristics in the cohort of preterm births and at term multiple births. Lombardy, Italy, 2007-2017.

|  | Reference At Term Births (N=5,283) | PTB (N=7,952) | p-value |
| --- | --- | --- | --- |
| *Year of birth* | |  | |
| 2007 | 402 (7.6) | 570 (7.2) | 0.5333 |
| 2008 | 465 (8.8) | 670 (8.4) |  |
| 2009 | 491 (9.3) | 733 (9.2) |  |
| 2010 | 485 (9.2) | 697 (8.8) |  |
| 2011 | 496 (9.4) | 777 (9.8) |  |
| 2012 | 504 (9.5) | 738 (9.3) |  |
| 2013 | 528 (10.0) | 810 (10.2) |  |
| 2014 | 484 (9.2) | 820 (10.3) |  |
| 2015 | 504 (9.5) | 714 (9.0) |  |
| 2016 | 492 (9.3) | 736 (9.3) |  |
| 2017 | 432 (8.2) | 687 (8.6) |  |
| *Maternal age (year****s*)** | |  | |
| <20 | 18 (0.3) | 42 (0.53) | <0.0001 |
| 20-24 | 124 (2.4) | 254 (3.2) |  |
| 25-29 | 704 (13.3) | 1,105 (13.9) |  |
| 30-34 | 1,921 (36.4) | 2,668 (33.6) |  |
| 35-39 | 2,030 (38.4) | 2,972 (37.4) |  |
| ≥40 | 486 (9.2) | 911 (11.5) |  |
| *Mean age (year****s)*** | 34.25 4.96 | 34.29 5.39 | <0.0001 |
| *Maternal citizenship* | |  | |
| Italian | 4,459 (84.4) | 6,756 (85.0) | 0.3829 |
| Not Italian | 824 (15.6) | 1,196 (15.0) |  |
| *Marital status* ^a^ | |  | |
| Married | 3,781 (73.6) | 5,514 (71.1) | 0.0018 |
| Not married | 1,357 (26.4) | 2,245 (28.9) |  |
| *Maternal education* ^b^ | |  | |
| Middle school | 1,099 (20.8) | 1,741 (22.0) | 0.1714 |
| High school | 2,404 (45.6) | 3,497 (44.1) |  |
| University | 1,770 (33.6) | 2,691 (33.9) |  |
| *Maternal employment* | |  | |
| Employed | 4,170 (78.9) | 6,291 (79.1) | 0.8035 |
| Not employed | 1,113 (21.1) | 1,661 (20.9) |  |
| *Mode of conception* | |  | |
| Spontaneous | 3,802 (72.0) | 5,603 (70.5) | 0.0613 |
| Non spontaneous | 1,481 (28.0) | 2,349 (29.5) |  |
| *Diabetes* |  |  |  |
| No | 5,240 (99.2) | 7,868 (98.9) | 0.1612 |
| Yes | 43 (0.8) | 84 (1.1) |  |
| *Hypertension* |  |  |  |
| No | 5,190 (98.2) | 7,724 (97.1) | <0.0001 |
| Yes | 93 (1.8) | 228 (2.9) |  |

^a^ Not included 338 missing data ^b^ Not included 33 missing data

**Table S3.** Population attributable fraction (PAF) of preterm birth (PTB) for advanced maternal according to calendar years. Lombardy, Italy, 2007-2017.

| *Calendar year* | *PAF (%)* |
| --- | --- |
| 2007 | 6.6 |
| 2008 | 6.6 |
| 2009 | 6.9 |
| 2010 | 9.0 |
| 2011 | 6.8 |
| 2012 | 9.2 |
| 2013 | 9.7 |
| 2014 | 12.0 |
| 2015 | 11.9 |
| 2016 | 12.2 |
| 2017 | 12.2 |

**Figure S1.** Percentage of preterm birth among singletons. Lombardy, Italy, 2007-2017.

**Figure S2.** Percentage of preterm birth among multiples. Lombardy, Italy. 2007-2017.
